# Supplementary material for: Control of visual adaptation depends upon task
Source: PLoS One. 2020 Feb 27;15(2):e0229343. doi: 10.1371/journal.pone.0229343 (PMC7046198; doi:10.1371/journal.pone.0229343)
Supplement: Attachment — Submitted filename: ResponseToReviews.docx [file pone.0229343.s001.docx]

**Response to reviews: Adaptation Is Weaker When It Harms Task Performance**

We thank both reviewers and the editor for their helpful comments. We have addressed each one carefully, and believe that the manuscript is much stronger as a result. Below, we address the concerns point by point.

**Reviewer #1:**

We thank the reviewer for the close read and thoughtful response.

**Comment:** “1. Line 44: It's true that threshold elevation after contrast adaptation is substantial, but a bit of an exaggeration to say that "a high contrast grating can cause detection thresholds for similar gratings to increase by orders of magnitude (e.g., Blakemore & Campbell, 1969)." I can't recall any studies finding more than about a 5x increase in threshold, even with high contrast and long duration - ie. less than 1 order of magnitude.

**Response:** We thank the reviewer for pointing this out, and have toned down the wording. The revised manuscript now reads (near line 43):

…exposure to a high contrast grating can cause detection thresholds for similar gratings to more than double.

**Comment:** “2. Line 91: How were head position and head orientation controlled? In an experiment on tilt aftereffects, this is important.”

**Response:** Head position and orientation were stabilized with a chin-rest. We apologize for the oversight and now report this in the revised manuscript.

**Comment:** “3. Was the display gamma-corrected? What was its mean luminance?”

**Response:** Yes both were done. We now report that (near line 91):

Mean display luminance was 42 candelas/meter^2^; presented luminances were measured with a PhotoResearch PR-655 and the displayed levels were linearized using software look-up tables.

**Comment:** “4. Line 96-107: Somewhere, need to state both the spatial spread constant (sigma) of the adapting Gabor, and the characteristics (e.g. SF, sigma, duration) of the test Gabor.”

**Response:** We thank the reviewer again for noticing our oversight. Sigmas for all Gabors were 1/6 of the stimulus size, and all test durations were 100 msec. We now report these in the revised manuscript.

**Comment:** “5. The illustration of timing in Fig 1B is very confusing: it appears to show the TAE test stimulus being shown immediately after the two secondary task intervals - but the text clearly implies that TAE and seondary stimuli were presented on separate, alternating trials of 1.5s each. Presumably Fig 1B is trying to save space by showing the two kinds of trials in a single sequence - but for the reader this really does not work. It might be clearer if the 4 kinds of trials (secondary vs TAE) x (baseline vs adaptation) were shown separately & clearly labelled, including an indication of when responses were made. The text on its own (p.6) is pretty clear, but Fig 1B creates confusion.

6. Figure 2 is even more mystifying. How the sequence of trials unfolds over time is completely unclear to me. I can't even tell which way the time axis goes (down, up sideways ? Or maybe all three ?). There must be a better way of picturing all this...And better labelling. For example, it is very unclear that the label 'Practice' applies not only to the right-hand column of boxes (close to the label) but also to the left-hand column (rather remote). The authors should try to put themselves in the mind of the reader who is not already highly familiar with the procedure...”

**Response:** We completely agree that the old methods figures were confusing, and we have revised them substantially, aiming for simplicity and comprehensibility. Please see revised Figures 1, 2, & 3.

**Comment: “**7. Line 220 'Figure 2' should be 'Figure 3'”

**Response:** Corrected.

**Comment: “**8. Experiment 1: I think there are at least two very significant shortcomings in the results, that relate to both the method used and the way the results are presented.

(i) First, for Fig. 3 (left) it is stated that "Plotted points are estimates of the orientation of the plaid components that appeared square, computed as the average presented orientation on each trial". They are 'estimates', yes, but it seems to me that they are quite likely to be biased ones. With this method of adjustment, the observer is trying to adjust the plaid (over trials) to appear 'square', but during this time the plaid's appearance is changing as the aftereffect decays. Thus there is no way to tell from the data which stimulus presentations might be close to the perceptual null point ('squareness') and which ones are in transition, thus sitting below or above the null point. Perhaps the authors are assuming that these estimation errors will average out, but that seems to me a fairly hazardous assumption, especially when we find out that the effect of interest (a task-dependent difference in the TAE) is really quite small.”

**Response:** This is point is well taken. To address concerns that averaging the presented orientation is a biased estimate of the point of subjective equality (orientations that produce square checks- neither too tall nor too short) we performed a simulation study. We assumed that simulated observers' true aftereffects decayed exponentially: They had an internal variable that corresponded to their PSE, the orientations that would appear square to them, and this decreased exponentially over time following the rate of our average real observer. For the simulated observers we also used a simple decision rule to determine response, thresholding the difference between the presented stimulus and the value of the PSE variable. Using these rules we simulated 500 observers in our experiment, and found that the running average of presented orientation just slightly overestimated the value of the observers' PSE. We attach a figure showing the results below, and now report the main findings of this simulation in the revised manuscript (near line 181):

TAE results were plotted as estimates of the orientation of the plaid components that appeared square. These were computed as the average presented orientation on each trial. Because the presented orientation was determined by a staircase-like procedure (based on the participants’ responses that attempted to make the checks appear square; see Tasks above), it is possible that simply averaging the presented orientations would produce a biased estimate. To test whether simple averaging of the staircase levels was a reasonable estimate, we performed a Monte-Carlo simulation of our experiment with a model observer responding. The model observer’s orientation that appeared square started at 48 degrees and decayed exponentially over time with a time constant 0.03, which produced a “true” effect similar to those seen in our and other studies. On each simulated trial, noise was added to the presented orientation, and the observer used a simple multiple threshold decision model to pick its response. Averaging across 10,000 simulations, the estimate obtained from simple averaging of the presented orientation fell close to the model observer’s true effect and did not reliably differ from it.

Reviewer Figure 1: Results of Monte Carlo simulation. The estimate obtained from simple averaging of the presented orientations across 10,000 runs is shown in black, and it falls close to the “true” adaptation decay curve used in the model observer. The gray region indicates plus/minus one standard deviation of the presented orientation.

**Comment:** “(ii) Second, it is stated (L. 227-230) that "To account for across subject differences in baseline (the plaid configuration that appeared square without adaptation), we computed a net TAE by subtracting baseline from adaptation for each observer. " This is a key point, because it is clear from the data (Fig 3) that the baseline the authors subtract is itself task-dependent, and not simply the average baseline over the two tasks. And close inspection of the TAE results (Fig 3) strongly suggests that the main difference in TAE for the two secondary tasks arises from the baseline data, not from the adaptation condition. This aspect of the data seriously undermines the authors' interpretation, that there was ‘stronger adaptation in the Fixation condition than in the Grating condition. (line 232-3)’.”

**Response:** This is a good point, and the possibility was raised in our original manuscript, and served as the motivation for Exp. 2. We note that while it is possible that the baseline differences can be interpreted differently, there is also an interpretation favorable to our hypothesis: It could be that the lower baseline requires larger adaptation effects to produce the observed lack of difference. We now discuss this more completely in the revised manuscript (near line 286):

One complication in the data is that performance on the plaid task differed as a function of task during the baseline blocks. Differential effects of attention in the two conditions may explain these differences. When participants attend the gratings in the secondary task display, they may have produced the small amount of contrast adaptation that was measured by the TAE task. This effect may have been reduced in the Fixation Task, when subjects did not attend the gratings. Such an effect, however, should also arise both at baseline and during the adaptation blocks; in this case subtracting the two would cancel it out. It remains possible, however, that the baseline differences were due to unrelated factors, such as noise of some sort, in which case they may explain, all by themselves, the differences in total TAE observed between tasks. Accordingly, we designed Experiment 2 to minimize possible differences in baseline performance.

**Comment:** “9. Experiment 2: The design of this experiment was tidied up in several ways, compared with Expt 1. The overall result was that there was now no significant effect of task on the TAE (p<0.16, Fig 6). There was, however, a significant effect for the first half of the experiment (Fig. 7). Unfortunately the baseline results for this subset of data are not presented, so we cannot tell what contribution any baseline difference may have made to the effect of task on TAE.”

**Response:** We now report results more completely for the analysis where they were broken down by day, and show baseline effects for both days in the relevant Figures. While the Exp. 2 day 1 results do contain a small baseline difference, the difference in adaptation is statistically significant even without subtracting the baseline out. This suggests that task indeed does have an effect on adaptation. We report these results in the revised manuscript (near line 333):

In the first session, total adaptation was reliably weaker when subjects performed the Grating task than when they performed the Fixation task (Figure 8A; p < 0.01, non-parametric signed-rank test used because of non-normal distribution of data). The difference was not reliable in the second testing session (Figure 8B; p > 0.5). In addition, while there was a small baseline difference between tasks in the first testing session, the difference in total adaptation was reliable even without correcting for baseline (p < 0.02, non-parametric signed-rank test).

**Comment:** “10. Discussion is interesting & wide-ranging, but I think it cannot overcome the basic weakness, or at the very least fragility, of the results. The case that "the processes controlling adaptation are sensitive to the task the observer is performing" is not convincingly supported.”

**Response:** Hopefully the response to point 9 has allayed the reviewers concerns somewhat. We now acknowledge the possible fragility of effects in the revised manuscript. We believe that they are worth reporting nevertheless, since they make an important, and to our knowledge novel, theoretical point (near line 420):

…the effect of task on adaptation was seen primarily during the first testing session. Our account of why this may be so- that participants learned to perform the secondary task independently of the effects of adaptation- is speculative. It additionally suggests that, in some circumstances, adaptation may not need to be regulated by task. Finally, relative to overall effects of adaptation, its modulation by task was not large in absolute terms. Characterizing when task performance can affect adaptation, and by how much, including in natural viewing during natural tasks, is an important line for future research. Nevertheless, our results provide reasonably strong support that task can affect the strength of adaptation.

**Reviewer #2:**

We thank the reviewer for the helpful comments.

**Comment:** “1. Throughout the authors assumed that adaptation made performance in the spatial frequency discrimination more “challenging” or “difficult” than the luminance discrimination but provide no data to support this claim. I would encourage them to present discrimination thresholds for both tasks before and after adaptation to substantiate the central tenet of their hypothesis.”

**Response:** We presented performance on the secondary tasks in Figures 5 and 8 in the original manuscript, but did not discuss these results very extensively. These are new Figures 6 and 9 in the revised manuscript, and we have added discussion of them:

(near line 269) Finally, we examined whether our assumption, that performance on the Grating task would be more challenged by adaptation than performance on the fixation task, was reflected in the data. Because adaptation was strongest in the first 40 sec of testing, we computed average performance over that interval and the two successive 40 sec periods. Performance on the Grating task was reliably reduced following adaptation during this first interval (Figure 6; t(8) = 2.8, p < 0.03). Performance was reduced numerically, but not reduced reliably for the Fixation task (t(8) = 1.3 p > 0.2), and the difference between the two tasks was not reliable (p> 0.5)….

(near line 342) We again analyzed the performance on the secondary tasks, before and after adaptation (Figure 9). Performance on the Grating task was again reliably reduced following adaptation during the first interval (t(8) = 3.9, p < 0.01). Performance was reduced numerically, but not reliably for the Fixation task (p > 0.5). The difference between the two tasks was not reliable (t(8) = 1.9, p < 0.09).

**Comment: “**76: ‘Our sample size was within the range of that used in the prior literature on contrast adaptation.’ Wouldn’t a statistically power analysis of the required sample size be more appropriate here rather than just doing what other studies have done before?”

**Response:** We agree that a power analysis would have been preferable, but because our paradigm was novel, we did not have good a priori estimates of our potential effect size (i.e. the difference in TAE between task conditions) and so we simply used sample sizes from the prior literature generally.

**Comment: “**86: How did they ensure a linear luminance (Gamma) correction on an LCD display (MacBook pro)?”

**Response:** We apologize for the oversight in reporting. We measured the display with a spectrophotometer and linearized it in software look-up tables. This has proven stable for LCD displays in our lab in general. We now report this in the revised manuscript:

Mean display luminance was 42 candelas/meter^2^; presented luminances were measured with a PhotoResearch PR-655 and the displayed levels were linearized using software look-up tables.

**Comment:** “89: typo - “Apparatus””

**Response:** Fixed
